# Amino Acids From Root Exudates Induce *Bacillus* Spore Germination to Enhance Root Colonisation and Plant Growth Promotion

**DOI:** 10.1111/1751-7915.70172

**Published:** 2025-05-30

**Authors:** Lili Tao, Xinli Sun, Pascale B. Beauregard, Taimeng Tan, Yuling Zhang, Jiyu Xie, Guidong Huang, Nan Zhang, Youzhi Miao, Qirong Shen, Zhihui Xu, Ruifu Zhang

**Affiliations:** ^1^ Jiangsu Provincial Key Lab of Solid Organic Waste Utilization, Jiangsu Collaborative Innovation Center of Solid Organic Wastes, Jiangsu Provincial Key Laboratory of Coastal Saline Soil Resources Utilization and Ecological Conservation, Educational Ministry Engineering Center of Resource‐Saving Fertilizers Nanjing Agricultural University Nanjing Jiangsu China; ^2^ Département de Biologie Université de Sherbrooke Sherbrooke Quebec Canada; ^3^ Department of Food Science Foshan University Foshan China

**Keywords:** amino acid, *Bacillus*, germination receptors, root exudates, spore germination

## Abstract

Strains of *Bacillus* species, plant growth‐promoting rhizobacteria, have been commercialised as biofertilisers; they are ideal for this because these species form spores that can be stored stably for a long time. However, for these spores to exert their full beneficial effects, they must germinate. The specific germination signals in the rhizosphere, particularly those from plant root exudates, remain largely unknown. Here, we investigated the germination signals from different growth states of cucumber ( *Cucumis sativus* ) for spores of *Bacillus velezensis* SQR9 and *Bacillus subtilis* NCIB 3610. We identified the corresponding germination receptors and compared them biochemically between the *Bacillus* species. Larger plants better stimulated spore germination. Five amino acids—L‐isoleucine, L‐ornithine, L‐valine, L‐serine and β‐alanine were—identified as spore germination signals. Combined application of a mixture of these amino acids with bacterial spores markedly enhanced the cucumber growth‐promoting properties of *B. velezensis* SQR9. The germination receptor for these amino acids was GerA in both *Bacillus* species. Differences in spore germination efficiency between *B. subtilis* and *B. velezensis* may be attributable to variations in the GerA ligand‐recognition sites. Expression of GerA from *B. subtilis* NCIB 3610 in *B. velezensis* SQR9 enhanced the spore germination rate of the latter. Our study highlights the pivotal role of amino acids in regulating spore germination of *Bacillus* and subsequent plant root colonisation, emphasising their potential to enhance the efficacy of *Bacillus*‐based biofertilisers. Engineering of germination receptors is a promising approach to enhance the spore germination efficiency of biofertiliser strains.

## Graphical abstract

Amino acids in cucumber root exudates trigger *Bacillus* spore germination via GerA, enhancing root colonisation and plant growth. This study reveals key germinators and receptors, advancing targeted biofertiliser applications for improved agricultural sustainability.

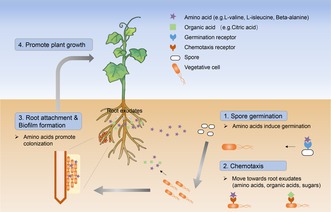

## 1 Introduction

Plant growth‐promoting rhizobacteria (PGPR) enhance plant growth by improving stress tolerance and supporting nutrient uptake (Bhattacharyya and Jha 2012; Borriss 2011; Lugtenberg and Kamilova 2009; Ling et al. 2010). Strains from the genera *Bacillus*, *Pseudomonas*, *Rhizobium* and *Azospirillum* have been commercialised as bioinoculants or biofertilisers, playing vital roles in boosting agricultural productivity (Verma et al. 2010; Ling et al. 2010; Vessey 2003; Bhattacharyya and Jha 2012; Bashan et al. 2014; Liu et al. 2024) Many PGPR strains lose viability after they are formulated into biofertilisers, resulting in a limited storage life and limiting their broader application (Vessey 2003). However, *Bacillus* species are particularly suitable for biofertiliser use because they form resilient endospores (Nicholson et al. 2000; Errington 2003; Piggot and Hilbert 2004; Borriss 2011). *Bacillus velezensis* SQR9 and *Bacillus subtilis* NCIB 3610 are two well‐studied *Bacillus* species used in biofertilisers, which can directly produce plant hormones, such as IAA, to regulate plant growth and development (Shao et al. 2015). Additionally, *B. velezensis* SQR9 can produce volatile compounds and protein bacillolysin (Fu et al. 2025) that stimulate root development by enhancing the expression of auxin biosynthesis and signalling in plants (Li et al. 2021). Furthermore, *Bacillus velezensis* SQR9 could influence the rhizosphere microbiomes by increasing the abundance of plant‐beneficial microorganisms, such as *Pseudomonas stutzeri* , that further promote plant health (Sun et al. 2022). Given these mechanisms, these *Bacillus* species hold great promise for applications in agricultural production. In China, over 70% of the microbial strains used in biofertilisers are from *Bacillus*. Even after 6 months of storage, these formulations retain a density of at least 2 × 10^7^ functional microorganisms per gram of fertiliser granule (Borriss 2011; Saxena et al. 2020; Raza et al. 2016). For these spores to exert their full beneficial effects, they must germinate. However, the specific germination signals in the rhizosphere remain largely unknown.

The signals that trigger spore germination are generally categorised into nutritional and non‐nutritional types. Nutritional signals include compounds such as sugars, purine nucleosides and combinations like asparagine, glucose, fructose and K^+^ (AGFK) (Xing and Harper 2020; Yasuda and Tochikubo 1997; Fan et al. 2023). Non‐nutritional signals involve factors such as Ca^2+^‐DPA (Dipicolinic acid), cationic surfactants like dodecylamine, salts, lysozyme, peptidoglycan fragments and physical triggers such as high pressure and moderately high temperature (Setlow 2003; Reineke et al. 2013). *B. subtilis* spores detect these signals through specific germination receptors encoded by the *gerA*, *gerB* and *gerK* operons (Moir 2023; Bremer et al. 2023). In *B. subtilis* , the GerA receptor has previously been reported to recognise L‐alanine; the GerB and GerK receptors collaboratively recognise AGFK (Setlow 2003). However, there has been limited research on the specific signals within the rhizosphere that trigger germination, particularly those from plant root exudates. *Bacillus* spores often remain dormant in soil, but they are more likely to germinate in the rhizosphere (Charron‐Lamoureux et al. 2020). For instance, in hydroponic systems, approximately 80% of spores transit to vegetative cells (Charron‐Lamoureux and Beauregard 2019). Understanding these root‐derived signals is critical for optimising the application and effectiveness of *Bacillus* spore‐based biofertilisers.

During different growth stages, plants release 11%–40% of their fixed carbon into the rhizosphere as root exudates, creating a nutrient‐rich environment that supports a dynamic microbial community (Bais et al. 2006; Jones et al. 2009; Sasse et al. 2018). These exudates include sugars, amino acids, organic acids, alcohols and aldehydes (Dakora and Phillips 2002; Bais et al. 2006). Their composition varies with the developmental stage of the plant, environmental conditions and the structure of the root‐associated microbiome (Badri and Vivanco 2009; Lareen et al. 2016). In addition to providing nutrients, root exudates play a key role in mediating plant–microorganism interactions, influencing microbial colonisation, competition and symbiosis (Bais et al. 2006; Haichar et al. 2014; Lareen et al. 2016). Thus, root exudates are crucial in fostering beneficial relationships between plants and microbes.

Exploring the communication mechanisms between plant roots and the spores of PGPR *Bacillus* species in the rhizosphere is a crucial step in understanding how these microorganisms exert their beneficial effects on plant growth. Previous studies have shown that *B. subtilis* and *B. velezensis* predominantly exist as vegetative cells in the rhizosphere but take on a spore form in the surrounding soil. *B. subtilis* NCIB 3610 and *B. velezensis* SQR9 are a group of non‐symbiotic PGPR strains that closely interact with plants, making them ideal candidates for studying spore germination during plant–microbe interactions. Based on these observations, we hypothesise that plants communicate with microorganisms through root exudates, which in turn trigger spore germination in *Bacillus* species. To test this hypothesis, we selected cucumber ( *Cucumis sativus* ) root exudates from different growth stages, along with spores of *B. velezensis* SQR9 and *B. subtilis* NCIB 3610, as experimental materials. Our results show that root exudates from all four growth stages effectively induce spore germination in both *B. velezensis* and *B. subtilis* , although germination rates vary between the two species. Furthermore, we identified 38 compounds in the root exudates and highlighted 5 amino acids as key germination signals. When combined with spores, these amino acids enhanced the plant growth‐promoting properties of *B. velezensis* SQR9. Additionally, we confirmed that the GerA receptor is responsible for spore germination in response to these amino acids. The observed differences in germination efficiency between *B. subtilis* and *B. velezensis* may be attributed to variations in the ligand‐binding sites of the GerA receptor. This study provides valuable insights into the interaction between *Bacillus* and plants, confirming the presence of spore germination‐inducing substances in root exudates. Engineering or mutating the germination receptor represents a promising approach to enhancing spore germination efficiency and improving agricultural productivity.

## 2 Experimental Procedures

### 2.1 Strains and Spore Preparation

Strains of *B. velezensis* SQR9 (China General Microbiology Culture Collection Center [CGMCC] accession no. 5808) and *B. subtilis* NCIB 3610 (Ramírez‐Guadiana et al. 2017) used in this study are listed in Table S1. *B. velezensis* SQR9 was used as the parental (wild‐type) strain for all gene‐deletion mutants.

To prepare spores, *B. velezensis* and *B. subtilis* cells were cultured in liquid 2× Schaeffer's‐glucose sporulation medium (Huo et al. 2012) at 37°C with shaking at 170 rpm. After incubating for 48 h, cells were harvested and centrifuged at 9000 g for 5 min. After removal of the supernatant, the resulting cells were resuspended in sterile water. The process was repeated three times to eliminate residual medium. The cell suspensions were then heat‐treated at 80°C for 20 min to kill vegetative cells, yielding suspensions containing ≥ 98% spores. The spore suspensions were centrifuged at 9000 g for 5 min, the supernatant was removed, and the resulting spores were stored at 4°C until use.

### 2.2 Plant Growth Conditions and Root Exudate Collection

Cucumber plants were cultured in 50‐mL sterile flasks containing liquid sucrose‐free ¼ MS medium (Feng et al. 2019) at 28°C with a 16‐h light/8‐h dark photoperiod. Root exudates were collected at the 1–4‐leaf stages (T1–T4). For exudate collection, roots were gently washed four times with sterile water to remove any residual growth medium. Plants were then transferred to fresh sterile flasks containing 50 mL of sterile water and cultured for an additional 24 h. After removing the plants, the root exudate solutions were filtered through a 0.22‐μm membrane (Millipore). To verify the sterility, 100 μL of the filtrate was plated on Luria‐Bertani (including tryptone, yeast extract, NaCl) agar medium plates and incubated overnight at 30°C to verify sterility. Sterile and filtered root exudates were lyophilized and stored at −80°C until use. The amounts of collected root exudates were recorded along with their corresponding root weights. Subsequently, the concentrations of root exudates were normalised by root weight to ensure consistent exudate quantities per unit root mass. This normalisation procedure guaranteed that the experimental treatments maintained equivalent root exudate concentrations across different plant growth stages.

### 2.3 Spore Germination Assay

Before the germination assay, spores were suspended in germination buffer (200 mM potassium phosphate buffer, pH 7.4) at OD_600 nm_ = 2 and heat‐treated at 65°C for 30 min followed by cooling on ice; this heat–ice pretreatment has been shown to promote germination (Broussolle et al. 2008). Germinant solutions were prepared by dissolving individual components of root exudates in sterile water to a final concentration of 10 mM. These solutions were filter sterilised using 0.22‐μm membranes (Millipore) before use. Pretreated spores were mixed with germinator solutions at a 1:1 ratio (final OD_600_ = 1.0). For the germination assay, the mixture was incubated at 37°C for 120 min then heat‐treated at 80°C for 20 min to inactivate germinated cells. The samples were serially diluted and plated on LB‐agar both before and after this heat treatment; the plates were incubated overnight at 37°C. Germination rates were determined by counting colony‐forming units (CFU) of total spores (before heat treatment) and ungerminated spores (after heat treatment). Phosphate‐buffered saline was used as the negative control for the germinant solution. All germination assays were performed in triplicate with independent spore batches.

### 2.4 Construction of Mutant and Replacement Strains

Spore germination receptor gene knockout mutants were constructed following the method described by Zhou et al. (2017). Briefly, the left flanking (LF) region (~1000 bp), direct repeat (DR) sequence (~500 bp) and right flanking (RF) region (~1000 bp) were amplified from the genomic DNA of *B. velezensis* strain SQR9 using primer pairs LF‐F/LF‐R, DR‐F/DR‐R and RF‐F/RF‐R, respectively. The primers used in this study are listed in Table S2. The PS cassette (~2300 bp; encoding the spectinomycin resistance gene) was amplified from p7S6 (Zhou et al. 2017) using primers PS‐F and PS‐R. These four fragments—LF, DR, the PS cassette and RF—were fused using overlap PCR. The fused products were transformed into competent cells of *B. velezensis* SQR9, and transformants were selected on Luria‐Bertani agar plates containing 100 mg/L spectinomycin. Final mutants were screened on Luria‐Bertani agar plates supplemented with 10 mM DL‐4‐chlorophenylalanine, and the deletions were verified by PCR using primer pair VF/VR.

For gene replacement, the upstream and downstream flanking regions of *gerA* were amplified from genomic DNA of *B. velezensis* strain SQR9, the spectinomycin resistance gene was amplified from plasmid p7S6, and the *gerA NCIB*_3610_ gene was amplified from genomic DNA of *B. subtilis* strain NCIB 3610. These three fragments were fused using overlap PCR and transformed into competent cells of *B. velezensis* SQR9. The substitution mutants were selected on LB‐agar plates containing 100 mg/L spectinomycin, and replacement was confirmed by PCR using primer pair VF/VR.

### 2.5 Pot Experiments

Cucumber seeds (Jinchun 4) were surface disinfected by immersion in 2% sodium hypochlorite and 75% ethanol, followed by multiple washes with sterile water. The sterilised seeds were then germinated in sterile conditions. Cucumber plants were initially grown under sterile conditions in the laboratory until the four‐leaf stage, with a cultivation period of over 20 days. Following this, the plants were transplanted into sterile soil and subjected to various treatments. The soil type is yellow‐brown soil; the collection site (119.180834°, 31.613530°) is BaiMa Experimental Station; the location is Nanjing Agricultural University, Jiangsu Province, China. Soils for the pot experiments were collected from a historically cucumber‐cultivated field with the following properties: pH 6.4, organic matter 24.5 g/kg, available N 166.3 mg/kg, available P 123.4 mg/kg, available K 211.4 mg/kg, total N 1.7 g/kg, total P 1.6 g/kg and total K 12.8 g/kg. The soil was used after being sterilised with gamma radiation (60 kGy) to ensure it was aseptic (Zhang et al. 2022). Four treatments were applied to the seedlings: CK, Mix, Spore and Spore with Mix. CK (controls) received sterile water. Plants in the Mix group were treated with a mixture of L‐isoleucine, L‐serine, L‐valine, β‐alanine and L‐ornithine at a concentration of 10 mM. Spore treatment involved inoculation with *B. velezensis* SQR9 spores (final concentration 10^7^ CFU/mL). Plants subject to Spore with Mix treatment received both *B. velezensis* SQR9 spores and the mixture of amino acids. Shoot dry weight, shoot fresh weight and shoot height of plants were measured after 21 days.

### 2.6 Colonisation Experiment

Root colonisation of *B. velezensis* SQR9 was measured 1, 7, 14 and 21 days post‐treatment in pot experiments. The plant roots were carefully excised and thoroughly washed to remove surface debris. After recording the fresh weight, the roots were immersed in PBS buffer and subjected to vortex mixing to detach rhizosphere bacteria. The suspension was then processed with intermittent ultrasonication (30 s of sonication followed by 20 s of rest, repeated for 6 cycles) to ensure complete bacterial detachment while maintaining cell viability. Following homogenisation, the original suspension was serially diluted in sterile PBS and plated onto appropriate culture media. Bacterial populations were subsequently quantified by counting CFU after incubation.

### 2.7 Prediction of Germination Receptor Protein β‐Alanine‐Binding Sites

Protein sequences of germination receptors from *B. velezensis* SQR9 and *B. subtilis* NCIB 3610 were downloaded from the NCBI and Ensembl databases. The sequences were compared using DNAMAN software with default parameters. Protein structure predictions were performed using the AlphaFold2 platform (available at: https://colab.research.google.com/github/sokrypton/ColabFold/blob/main/AlphaFold2.ipynb#scrollTo=_sztQyz29DIC). The structures of β‐alanine (CAS: 107‐95‐9) were obtained from the ChemSpider platform (ChemSpider: Search and Share Chemistry—Homepage). Structural comparison and molecular docking analyses were carried out using PyMOL 2.5.5 software. Structural similarity was assessed by calculating root‐mean‐square deviation (RMSD) values, and key binding residues within the protein binding pockets were identified. The binding energy was predicted using Autodock vina 1.2.7 (Feng et al. 2022; Trott and Olson 2010).

### 2.8 Data Analysis and Figure Generation

Statistical differences among treatments were evaluated using Duncan's multiple range test (*p* < 0.05) in SPSS software version 25.0 (IBM, Armonk, NY, USA). Figures were generated using GraphPad Prism version 9.0 and RStudio version 4.4.1, using the ggplot2 and ComplexHeatmap packages. Diagrams of Figure 6 were created using Biorender (www.biorender.com).

## 3 Results

### 3.1 Induction of Spore Germination by Cucumber Root Exudates Is Influenced by Plant Developmental Stage

**FIGURE 1:**
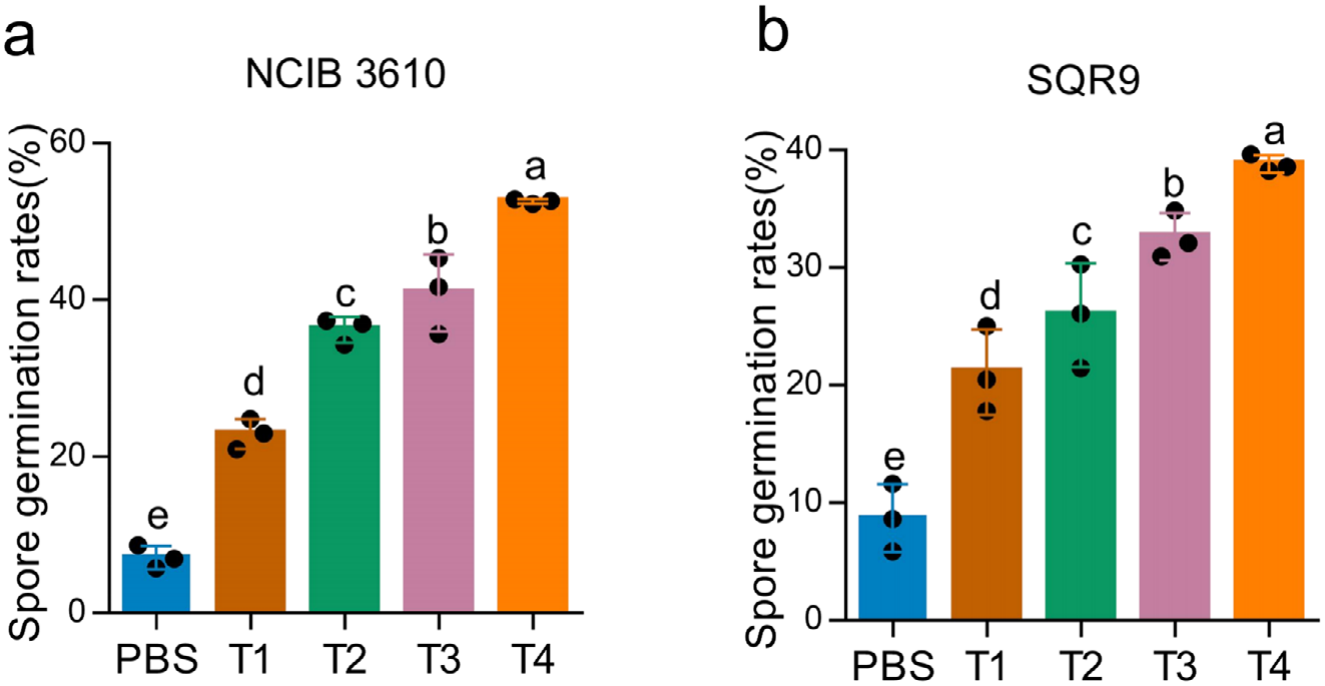
Spore germination is triggered by root exudates. Germination rates (%) of *Bacillus velezensis* SQR9 (a) and *Bacillus subtilis* NCIB3610 (b) spores in root exudates from four different growth stages. T1, T2, T3 and T4 correspond to root exudates collected when cucumber plants have one, two, three or four true leaves, respectively. Data presented are the percentage of germinated spores, shown as mean values + SD from three independent biological replicates. Different letters indicate statistically significant differences according to Duncan's multiple range tests (*p* < 0.05).

Because *Bacillus* spores first need to germinate in the rhizosphere to exert their beneficial functions on plants, we investigated how cucumber root exudates influence the germination of spores of *B. velezensis* SQR9 and *B. subtilis* NCIB 3610. The results showed that spores of *B. velezensis* SQR9 (Figure 1a) and *B. subtilis* NCIB 3610 (Figure 1b) were successfully germinated in the root exudates from all four cucumber growth stages. As cucumber growth progresses, the spore germination rates of both strains gradually increase. Notably, at each growth stage, *B. subtilis* NCIB 3610 consistently exhibits a higher germination rate than *B. velezensis* SQR9. Root exudates from the T4 stage exhibited the greatest germination effect; the germination rates of *B. subtilis* NCIB 3610 showed 52.6%, and *B. velezensis* SQR9 showed 38.81%. These results suggest that the root exudates contain molecules that promote *Bacillus* spore germination, with the T4 stage either providing a higher concentration of these germinators.

### 3.2 Amino Acids Were the Key Inducers of Spore Germination

**FIGURE 2:**
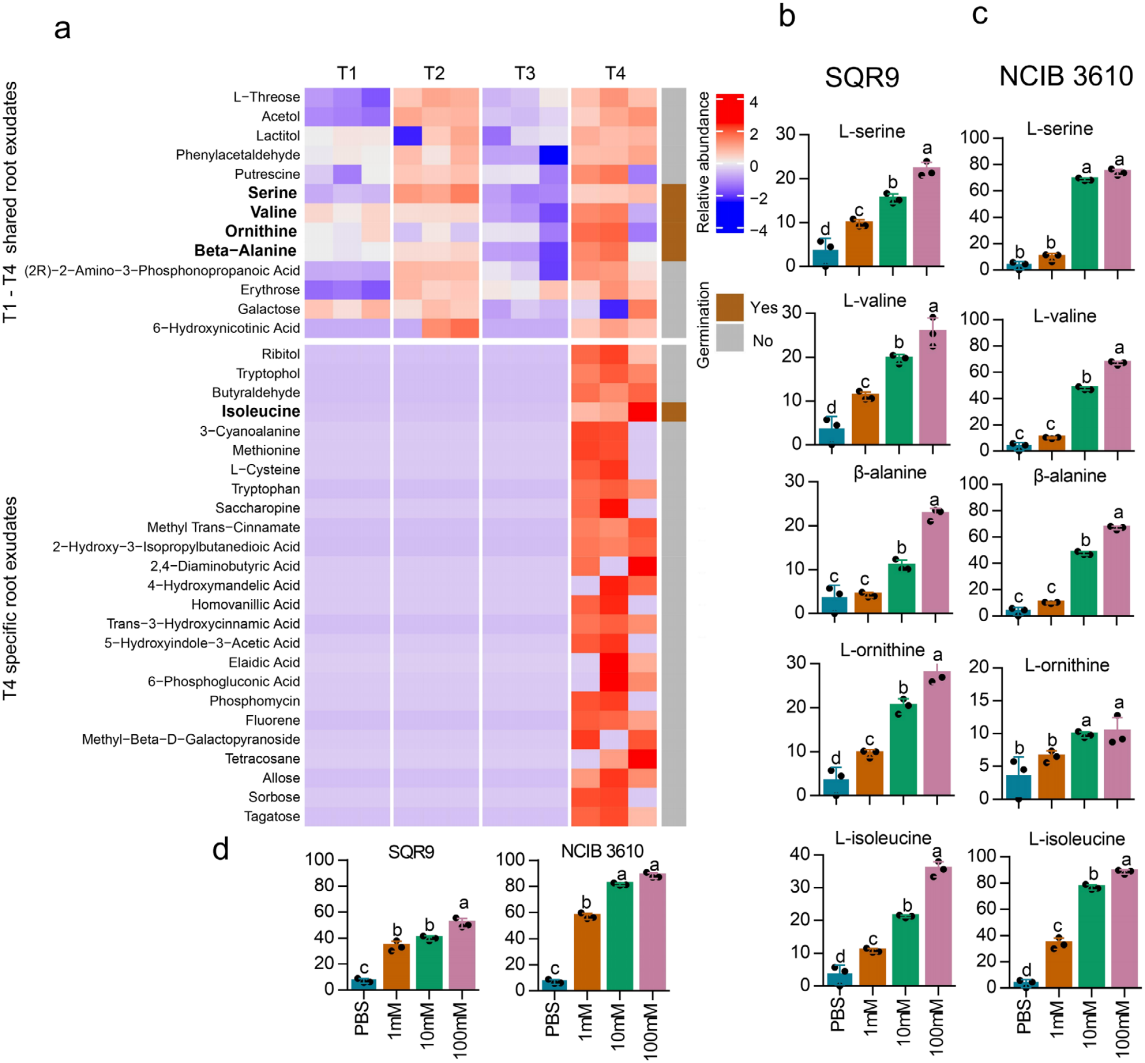
Spore germination of *B. velezensis* and *B. subtilis* was induced by specific root exudates. (a) The effect of specific root exudates on spore germination of *B. velezensis* SQR9. The left heatmap shows the relative abundance of compounds in root exudates from different stages. Data were derived from Feng et al. (2023). The right heatmap shows the ability to induce spore germination; the colour blue indicates no germination, and the colour red indicates germination. Spore germination rates of *B. velezensis* SQR9 (b) and *B. subtilis* NCIB 3610 (c) in different concentrations of amino acids. Spore germination rates of *B. velezensis* SQR9 and *B. subtilis* NCIB 3610 (d) in different concentrations of amino acids. PBS is set as the negative control. Data presented are mean + SD; different letters indicate significant differences according to Duncan's multiple range tests (*p* < 0.05).

Root exudates from the T4 stage of cucumber exhibited the highest spore germination rates for *B. velezensis* SQR9; we therefore assessed the compounds that are abundant or specifically present at this stage. The composition of cucumber root exudates at various growth stages has been determined previously (Feng et al. 2023). Among the components, 38 compounds were tested for their ability to induce spore germination of *B. velezensis* SQR9 (Figure 2a). Individually, five amino acids—L‐isoleucine, L‐serine, L‐valine, β‐alanine and L‐ornithine—were found to significantly induce spore germination. The germination‐inducing effect was increased with increasing concentrations of these amino acids (Figure 2b,c). Notably, L‐isoleucine, which was detected in cucumber root exudate only at the T4 stage, demonstrated the highest germination‐inducing effect (spore germination rate 35.64%). When combined, these five amino acids showed a germination effect comparable to or even higher than that of T4 root exudate (Figure 2d). Together, these findings suggest that amino acids in root exudates play a crucial role in triggering spore germination of *Bacillus* species.

### 3.3 GerA Is the Germination Receptor Responsible for Detecting Amino Acids

**FIGURE 3:**
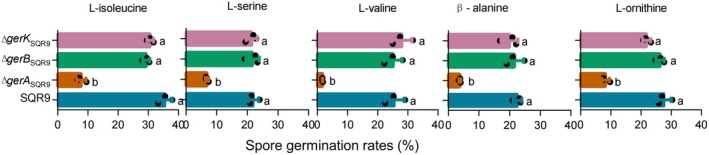
Spore germination of *B. velezensis* mutants and complementary strain amino acids. Spore germination of *B. velezensis* germination receptor mutants. Data presented are mean + SD, different letters indicate significant difference according to the Duncan's multiple range tests (*p* < 0.05).

Having identified amino acids as key inducers of spore germination for *B. velezensis* SQR9, we next sought to determine the specific germination receptors involved in this process. Currently, *B. velezensis* SQR9 has been developed into a commercial product in China, whereas strain NCIB 3610 remains at the research stage. Three distinct operons encoding germination receptors were identified in the genome of *B. velezensis* SQR9: the *gerA* operon (comprising *gerAA*, *gerAB* and *gerAC*), the *gerB* operon (*gerBA*, *gerBB* and *gerBC*) and the *gerK* operon (*gerKA*, *gerKB* and *gerKC*). Although the gene names follow a similar format (e.g., “A”, “B”, “C”), they represent distinct gene products specific to each operon. To identify the receptor(s) responsible for amino acid sensing, we constructed knockout mutants of *B. velezensis* SQR9 for each of these operons and assessed the ability of spores of the resulting cells to germinate in the presence of amino acids. The germination rates were decreased in the ∆*gerA* mutant compared with the wild‐type for all five amino acids (Figure 3). In contrast, the germination rates were unaffected in the ∆*gerB* and ∆*gerK* mutants. These results suggest that the GerA receptor is responsible for recognising L‐isoleucine, L‐serine, L‐valine, β‐alanine and L‐ornithine to induce spore germination in *B. velezensis* SQR9.

### 3.4 Exogenous Addition of Amino Acids Enhances the Plant Growth‐Promoting Ability and Root Colonisation of *B. velezensis* SQR9 Spores

**FIGURE 4:**
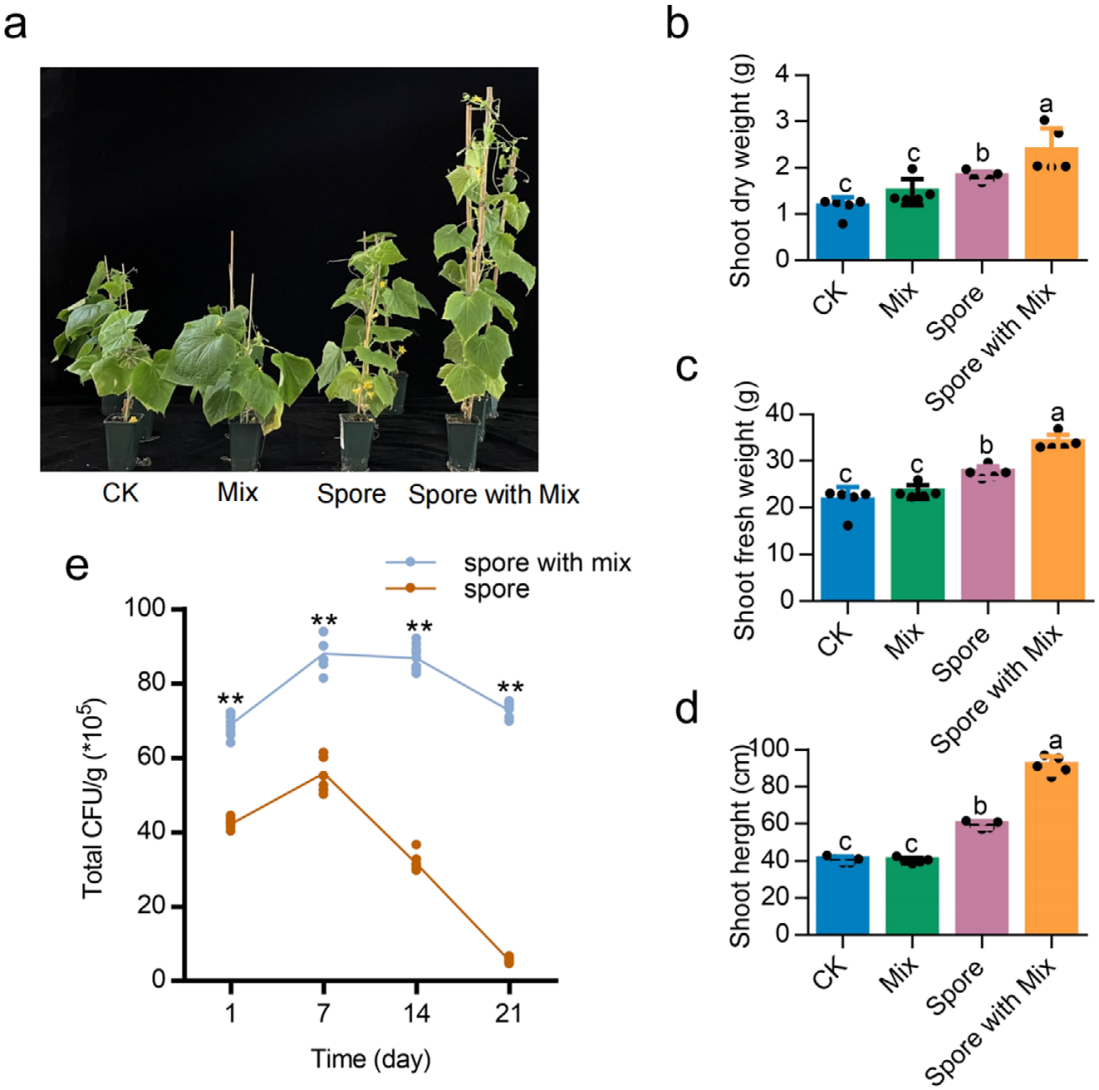
Amino acids enhance the plant growth‐promoting effect and root colonisation of *B. velezensis* SQR9 spores. (a) Plant phenotype. CK, seedlings are added with water; Mix, seedlings inoculated with 5 mL mix amino acid; Spore, seedlings inoculated with 5 mL bacterial spore suspensions (108 CFU mL^−1^); Spore with mix inoculated with 5 mL bacterial spore suspensions and mix amino acid. Three representative plants were shown. Shoot dry weight (b), shoot fresh weight (c) and shoot height (d) of the plants. Data presented are mean + SD, different letters (a, b, c) above the bars indicate significant difference according to the Duncan's multiple range tests (*p* < 0.05). *N* = 5. (e) Root colonisation of *B. velezensis* SQR9 in sterile soil conditions. Spore indicate the cells were inoculated as spores alone, Spore with Mix indicate the cells were inoculated as spores with mixture of amino acids (L‐isoleucine, L‐serine, L‐valine, ß‐alanine and L‐ ornithine). “**” indicates significant difference according to the test (*p* < 0.01).

Given that amino acids effectively induced spore germination, we investigated whether the exogenous addition of these amino acids could enhance the plant growth‐promoting effects of *B. velezensis* SQR9 in a sterile soil environment. Co‐inoculation of *B. velezensis* SQR9 spores with a mixture of amino acids significantly boosted plant growth compared with spores alone, with increases of 55.63% in plant height, 51.26% in dry weight and 36.60% in fresh weight (Figure 4a–d). Control treatments where only amino acids were applied to the rhizosphere had no significant plant growth‐promoting effect. These findings confirm that the enhanced plant growth is attributable to the germination of spores of *B. velezensis* SQR9, rather than to any nutritional effect from dead cells or amino acids.

The results of the colonisation experiment with *B. velezensis* SQR9 showed a peak in root colonisation at 7 days, followed by a decline, yet the exogenous addition of amino acids consistently enhanced colonisation at all time points compared with plants inoculated with spores alone (Figure 4e). These results collectively suggest that amino acids not only induce spore germination but also promote root colonisation by *B. velezensis* strain SQR9, thereby contributing to more effective plant growth promotion.

### 3.5 Substituting the GerA Receptor of *B. velezensis* SQR9 With That of *B. subtilis* NCIB 3610 Enhances Spore Germination

**FIGURE 5:**
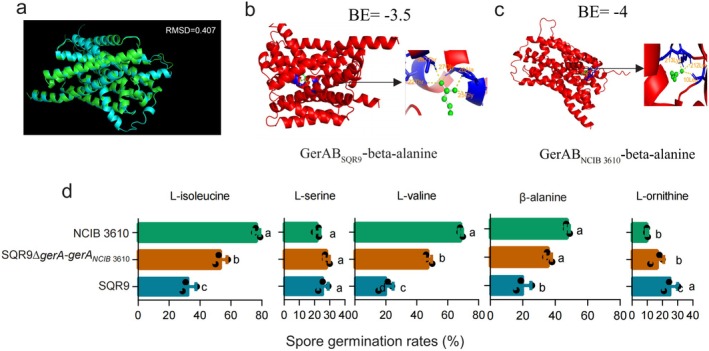
Structural and functional comparison of the GerA subunit in *Bacillus velezensis* SQR9 and *Bacillus subtilis* NCIB 3610. Protein structure comparison (a) of the B subunit of the GerA germination receptor from *B. velezensis* SQR9 and *B. subtilis* NCIB 3610. The green one was SQR9, and the blue one was NCIB 3610. RMSD showed 0.407, indicating high similarity. Red represents the protein structure, green represents the small molecule beta‐alanine, blue represents the binding site and yellow represents the hydrogen bond. A close‐up view of the amino acid‐binding pocket of the subdomain of B subunit of GerA from SQR9 (b) and from NCIB 3610 (c). Binding energy (BE) showed −3.5 kcal/mol (b) and −0.4 kcal/mol (c). (d) Spore germination of *B. velezensis* gerA complemented with GerA of *B. subtilis* NCIB 3610. Data presented are the percentage of germinated spores, shown as mean values + SD from three independent biological replicates. Different letters indicate statistically significant differences according to Duncan's multiple range tests (*p* < 0.05).

We observed that the germination rate of *B. subtilis* NCIB 3610 was 90% in a mixture of L‐isoleucine, L‐serine, L‐valine, β‐alanine and L‐ornithine at a concentration of 10 mM, while that of *B. velezensis* SQR9 was < 50%. We hypothesised that the recognition ability of GerA of strain *B. subtilis* NCIB 3610 is higher than that of GerA from strain *B. velezensis* SQR9. The ligand‐binding pocket of the receptor is located on GerAB (Artzi et al. 2021). The protein sequences and structures of GerAB of both strains were compared (Figure S1, Figure 5a–c). The proteins share 66.07% amino acid identity. Their structures also show high similarity (RMSD = 0.407A), yet the binding sites are different. In *B. subtilis* NCIB 3610, the binding site residues for β‐alanine predicted by the AlphaFold2 platform and PyMOL 2.5.5 software were Lys‐213, Lys‐212 and Leu‐10, whereas in *B. velezensis* SQR9, the binding site residues were Thr‐22, Thr‐287, Gly‐25, Ala‐26 and Gly‐27. To further assess binding affinity, we performed docking analysis using AutoDock. The predicted binding energy for the *B. velezensis* SQR9 receptor–ligand complex was −3.5 kcal/mol, whereas that for the *B. subtilis* NCIB 3610 receptor–ligand complex was −4 kcal/mol. The results demonstrate that the germination receptor of *B. subtilis* NCIB 3610 has a lower binding energy with β‐alanine, which may account for its higher germination rate compared to *B. velezensis* SQR9 in the rhizosphere. To explore whether the differences in the GerAB receptors lead to the difference in spore germination rates between *B. subtilis* NCIB 3610 and *B. velezensis* SQR9, we substituted GerA_SQR9_ with GerA_NCIB 3610_ in an engineered strain of *B. velezensis* . The resulting strain, *B. velezensis* SQR9 ∆*gerA*/*gerA*_NCIB 3610_, exhibited significantly higher germination rates in response to L‐isoleucine, L‐valine and β‐alanine than the wild‐type strain (Figure 5d). The exception was for L‐ornithine and L‐serine, which showed a low inducing effect on spore germination in *B. subtilis* NCIB 3610, potentially explaining the decreased germination observed in strain *B. velezensis* SQR9 ∆*gerA*/*gerA*_NCIB 3610_ compared with its wild‐type on stimulation with L‐isoleucine, L‐valine and β‐alanine.

## 4 Discussion

Spore germination rates are significantly higher in the rhizosphere than in the surrounding soil (Charron‐Lamoureux et al. 2020), suggesting the presence of specific substances in the rhizosphere that promote germination. In this study, we aimed to identify components of root exudates that contribute to spore germination. We discovered that root exudates from different stages of cucumber growth can effectively induce the spore germination of *Bacillus* species. The effective compounds were identified as amino acids: L‐isoleucine, L‐ornithine, L‐valine, L‐serine, and β‐alanine. The efficiency of germination varied depending on the *Bacillus* species, and genetic modification of germination receptors was found to affect spore germination rates. The exogenous addition of amino acids increased the colonisation of plant growth‐promoting *B. velezensis* .

Root colonisation by PGPR is essential for them to promote plant health and growth (Keshmirshekan et al. 2024; Shah et al. 2021). However, root colonisation is not the sole factor influencing plant growth (Charron‐Lamoureux et al. 2024). We have highlighted that rhizosphere microorganisms interact with plants through the release of metabolites. For instance, *Bacillus velezensis* SQR9 produces growth‐promoting substances like IAA, which directly enhance plant growth. Additionally, plants can influence microbial gene expression to further boost growth‐related compounds. However, other factors, such as plant genotype (Zhang et al. 2019), root exudates (Huang et al. 2019), pathogen presence (Wei et al. 2018) and microbial interactions in the rhizosphere (Liu et al. 2022), also play significant roles in shaping the rhizosphere microbiome and plant growth.

Root exudates are generally recognised as playing crucial roles in plant–microbe interactions within the rhizosphere, such as regulating chemotaxis, motility, biofilm formation and metabolism of soil microbes (Compant et al. 2010; Yuan et al. 2015; Bais et al. 2004; Zhang et al. 2014, 2015; Preece and Peñuelas 2016). For instance, 40 compounds in cucumber root exudates were identified as chemoattractants for *B. velezensis* strain SQR9, including organic acids, amino acids and sugars (Feng et al. 2018). Among them, D‐galactose also stimulates biofilm formation and the effect is not caused by direct growth promotion of *B. velezensis* SQR9 (Liu et al. 2020). Moreover, *B. velezensis* SQR9 can use plant polysaccharides as sources for synthesising biofilm matrix exopolysaccharides (Xu et al. 2019). Malic, fumaric, gluconic and glyceric acids, lysine, serine, alanine and mannose contribute to the overall recruitment of *B. velezensis* SQR9 to the rhizosphere (Feng et al. 2019). Bernier and colleagues demonstrated that these amino acids are also instrumental in biofilm formation by *Azotobacter chroococcum* (Palareti et al. 2016).

The composition of root exudates varies depending on the plant species, the age of the individual plant and external environmental factors (Haichar et al. 2014). Root exudates of young plants were reported to regulate genes involved in the biosynthesis of antibiotics in *B. velezensis* SQR9, while exudates from larger plants regulated genes related to plant growth promotion (Feng et al. 2023). It is important to note that our research was conducted under hydroponic conditions in a short‐term experiment. Future studies should consider exploring spore germination and plant growth dynamics under soil conditions, which will provide a more comprehensive understanding of the relationship between plant growth and microbial colonisation. It is essential to carefully manage the timing of biofertiliser application, particularly when using *Bacillus* as the functional microorganism, to maximise its effectiveness. The identified germinators L‐isoleucine, L‐serine, L‐valine, β‐alanine and L‐ornithine were also reported as chemoattractants for *B. velezensis* SQR9 (Feng et al. 2018). *B. subtilis* NCIB 3610 exhibits chemotaxis toward all 20 L‐amino acids, a process mediated by the McpC receptor (Glekas et al. 2012).

**FIGURE 6:**
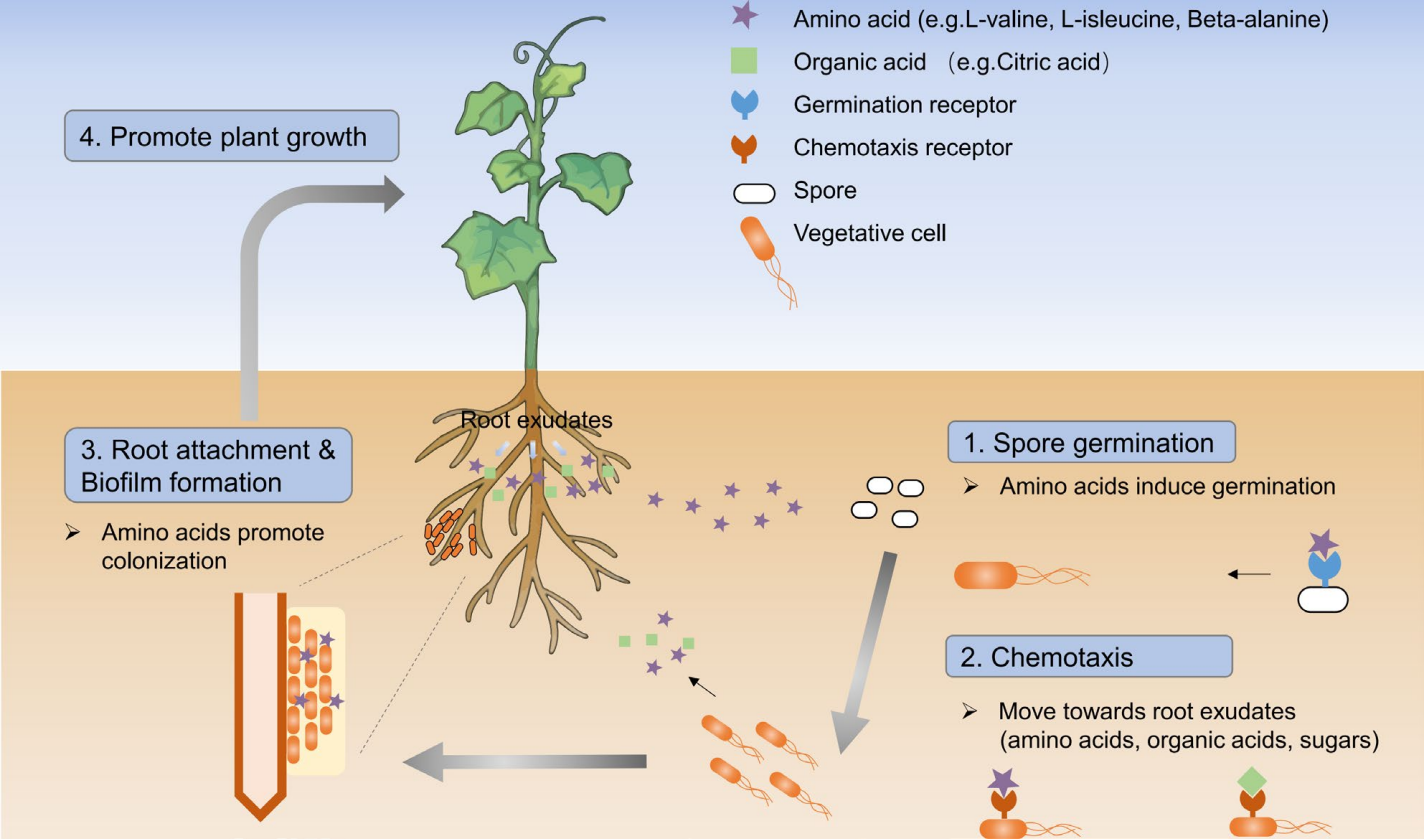
Schematic diagram illustrating multiple functions of amino acids in SQR9 root colonisation. The colonisation process involves spore germination, chemotaxis towards root exudates, root attachment, biofilm formation and ultimately, plant growth promotion.

This research sheds light on the multiple roles of amino acids in the rhizosphere, mediating plant–microbe interactions via different mechanisms and regulating microbial interactions. For instance, amino acid cross‐feeding contributed to synergistic biofilm formation of a *B. velezensis* – *P. stutzeri* consortium (Sun et al. 2022). This metabolic cooperation facilitated nutrient sharing, promoting biofilm stability and resilience, which is essential for the successful colonisation of plant roots and protection against pathogens (Sun et al. 2022). In the gastrointestinal system, amino acids mediate complex interactions between the host and microbial communities, modulating immune responses and metabolic adaptation to environmental changes (Zhang et al. 2024). Similarly, in the rhizosphere, amino acids from root exudates influence microbial community dynamics by attracting beneficial microbes, thereby enhancing plant health and soil fertility (Sasse et al. 2018). Such studies underscore the multifaceted roles of amino acids in shaping both microbial interactions and overall ecosystem function. The multifaceted roles that amino acids play in mediating plant–*Bacillus* interactions in the rhizosphere are summarised in Figure 6.

Our study also revealed significant differences in spore germination efficiency between two *Bacillus* species in response to amino acids. *B. subtilis* NCIB 3610 exhibited germination rates > 90%, while *B. velezensis* SQR9 showed rates < 50% when exposed to a mixture of amino acids. Previous studies have confirmed that heterogeneity in nutrient‐triggered spore germination among *Bacillus* species is influenced by various factors (van der Voort et al. 2010; Abee et al. 2011; Madslien et al. 2014; Broussolle et al. 2008). First, germination receptors in *Bacillus* species display different sensitivities in response to germinants. Second, the sensitivity can be influenced by external factors such as pH and sporulation conditions (Nguyen Thi Minh et al. 2011; Ramirez‐Peralta et al. 2012; Loøvdal et al. 2011; Brown et al. 1979). Third, the specificity of the receptors differs between species. In both *Bacillus* species studied here, β‐alanine recognition receptor was GerA (Figure 3). However, structural comparison of the GerA receptors of the two *Bacillus* species revealed differences in the ligand‐binding pockets, which may explain the observed differences in germination efficiency between the two species; these findings warrant further investigation. Based on their characteristics, we propose to modify the receptors to develop high affinity, as a novel strategy to improve germination efficiency. Spore germination in the rhizosphere is a critical process that allows *Bacillus* spores to exit dormancy, proliferate, and function effectively as biofertilisers. As the plant progresses into later developmental stages, its nutrient demands increase substantially, requiring the assistance of beneficial microbes to mobilise and activate otherwise inaccessible nutrients in the soil. Therefore, the germination process is essential for combating soil‐borne pathogens and supporting plant growth in agriculture (Bhattacharyya and Jha 2012; Xing and Harper 2020). However, despite their importance, effective stimulants specifically targeting spore germination remain limited. Substances that induce spore germination include L‐alanine, L‐valine, a mixture of AGFK, Ca^2+^‐DPA and certain cortex‐lytic enzymes (Setlow 2003; Fan et al. 2023). Our results suggest that the exogenous addition of amino acids could enhance the plant growth‐promoting properties and root colonisation of *B. velezensis* SQR9 spores. Compound liquid amino acids have been shown to enhance PGPR activity (Liu et al. 2016), providing plants with nutrition and resulting in improved quality and a shorter production cycle with increased dry material content (Wahba et al. 2015). Composting matured manure with amino acids before inoculating with *B. velezensis* SQR9 has been shown to increase the biomass of SQR9 in biofertilisers (Liu et al. 2016). Furthermore, the application of high‐quality, novel biofertilisers containing PGPR, especially when combined with amino acids, provides a valuable nitrogen source for plants; the emerging vegetative cells are attracted to the plant root, guided by chemotaxis receptors that detect amino acids in root exudates (Feng et al. 2018). Root exudates also stimulate biofilm formation and facilitate microbial colonisation (Zhang et al. 2014, 2015). Furthermore, root exudates regulate the expression of *B. velezensis* SQR9 genes involved in antibiotic biosynthesis and plant growth promotion, differentially depending on the developmental stage of the plant (Feng et al. 2023). The PGPR can enhance nutrient uptake and overall plant health (Bhattacharyya and Jha 2012). Beyond root exudates, other rhizosphere substrates, such as microbial metabolites, may also stimulate spore germination. In the future, the patterns of *Bacillus* spore germination will be studied in the context of tripartite interactions involving plant–microbe and microbe–microbe relationships.

## 5 Conclusion

Our study highlights the pivotal role of L‐isoleucine, L‐serine, L‐valine, β‐alanine and L‐ornithine in regulating spore germination of *Bacillus* species and subsequent plant root colonisation, emphasising their potential to enhance the efficacy of *Bacillus*‐based biofertilisers. GerA is a key receptor in *B. velezensis* responsible for sensing root exudates and initiating spore germination. The binding sites and affinity between the receptor of GerA and amino acids are among the critical factors influencing the spore germination efficiency of *B. velezensis* . By identifying key germinators and their corresponding receptors, this research deepens the understanding of the interactions between plants and beneficial *Bacillus*, paving the way for more targeted applications of biofertilisers.

## Author Contributions

**Lili Tao:** data curation, visualization, writing – original draft. **Xinli Sun:** methodology, writing – original draft. **Pascale B. Beauregard:** conceptualization, investigation. **Taimeng Tan:** investigation, data curation. **Yuling Zhang:** investigation, methodology. **Jiyu Xie:** investigation, formal analysis. **Guidong Huang:** funding acquisition, resources. **Nan Zhang:** investigation, visualization. **Youzhi Miao:** formal analysis, methodology. **Qirong Shen:** conceptualization, funding acquisition. **Zhihui Xu:** conceptualization, funding acquisition, writing – review and editing. **Ruifu Zhang:** conceptualization, supervision, resources.

## Conflicts of Interest

The authors declare no conflicts of interest.

## Supporting information


**Table S1:** *Bacillus* strains used in this study.
**Table S2:** Primers used for constructing and verifying strain of mutants and replenishment.
**Table S3:** All chemicals used in the study.
**Figure S1:** Sequence alignment comparison of the B subunit of the GerA germination receptor from *B. velezensis* SQR9 and *B. subtilis* NCIB 3610.

## Data Availability

The datasets used and analysed during the current study are available from the corresponding author on reasonable request.
