# Supplementary material for: Amino Acids From Root Exudates Induce Bacillus Spore Germination to Enhance Root Colonisation and Plant Growth Promotion
Source: Microb Biotechnol. 2025 May 30;18(6):e70172. doi: 10.1111/1751-7915.70172 (PMC12124999; doi:10.1111/1751-7915.70172)

**Supplemental Data**

Table. S1 *Bacillus* strains used in this study

| strain | Genotype | Source of reference |
| --- | --- | --- |
| SQR9 | Wild type | Zhang et al. 2014 |
| NCIB 3610 | Wild type | Ramírez-Guadiana et al. 2017 |
| SQR9 | Δ*gerA* | This study |
| SQR9 | Δ*gerB* | This study |
| SQR9 | Δ*gerK* | This study |
| SQR9 | ∆*gerA*/*gerA*_NCIB3610_ | This study |

Table. S2: Primers used for constructing and verifying strain of mutants and replenishment.

| Mutant | Primer name | Primer sequence (5’-3’) |
| --- | --- | --- |
| ∆gerA | LF-F | cgccgtcgggaatgtatcgt |
|  | LF-R | tctcccgagggacggggtcacgacagtcacccgccttcca |
|  | DR-F | tggaaggcgggtgactgtcgtgaccccgtccctcgggaga |
|  | DR-R | cgcagtttaaacaggcgtaataatatccggtgatcgaagcggg |
|  | PS-F | cccgcttcgatcaccggatattattacgcctgtttaaactgcg |
|  | PS-R | ccttgaattccgattgcgccccgttcgtataatgtatgct |
|  | RF-F | agcatacattatacgaacggggcgcaatcggaattcaagg |
|  | RF-R | gcaaacggcgagttgtcgac |
|  | VF | ttcaccgaactgcggatggg |
|  | VR | tcatacggcctgcattccatc |
|  |  |  |
| ∆*gerB* | LF-F | catatatccggtaggccgcc |
|  | LF-R | aaaaaatacgatgaaggctaaatgttccccccctttccat |
|  | DR-F | atggaaagggggggaacatttagccttcatcgtatttttt |
|  | DR-R | cgcagtttaaacaggcgtaataacggacatccttgagcactta |
|  | PS-F | taagtgctcaaggatgtccgttattacgcctgtttaaactgcg |
|  | PS-R | Agctccgcatgtatcgtcaaccgttcgtataatgtatgct |
|  | RF-F | agcatacattatacgaacggttgacgatacatgcggagct |
|  | RF-R | tcaacggtagccggcaccaa |
|  | VF | aaggttttgatggtgacgcct |
|  | VR | tgatgacctatgtctgcgcg |
|  |  |  |
| ∆*gerK* | LF-F | tcaccgctcatcgttaccgt |
|  | LF-R | cccggcgcaccggccgggtttcgcaaaaaacctttcctcg |
|  | DR-F | cgaggaaaggttttttgcgaaacccggccggtgcgccggg |
|  | DR-R | cgcagtttaaacaggcgtaataacgtaaacggctcaaagcgcg |
|  | PS-F | cgcgctttgagccgtttacgttattacgcctgtttaaactgcg |
|  | PS-R | tgcttaaaaaatggcggcatccgttcgtataatgtatgct |
|  | RF-F | agcatacattatacgaacggatgccgccattttttaagca |
|  | RF-R | gccgctccgtatgatatacgg |
|  | VF | aaggacaggacagcagcaat |
|  | VR | tctcctgatgatccggcctg |
|  |  |  |
| SQR9 ∆*gerA*/*gerA*_NCIB3610_ | LF-F | gtcggaatgcttgtgggcac |
|  | LF-R | cgttacgttattagttattgacgcggtcaccttattgg |
|  | DR-F | cgaataaggtgaccgcgtcaataactaataacgtaacg |
|  | DR-R | caaatattcaaaggattcccgcgcgcgtataatgtatgctata |
|  | Spc-F | tatagcatacattatacgcgcgggaatcctttgaatatttg |
|  | Spc-R | cttgggcgctgccattgttagtgaattaggcggctgctatttg |
|  | RF-F | caaatagcagccgcctaattcactaacaatggcagcgcccaag |
|  | RF-R | tcgcgcagaatccagtgtcg |
|  | VF | ccactggcgctgatttggtt |
|  | VR | agcggctccgaagtattcgc |

Table. S3: All chemicals used in the study

| Name | CAS | Company |
| --- | --- | --- |
| L-Threose | 95-44-3 | aladdin |
| Acetol | 116-09-6 | aladdin |
| Lactitol | 585-86-4 | aladdin |
| Phenylacetaldehyde | 122-78-1 | aladdin |
| Putrescine | 462-76-6 | aladdin |
| Serine | 56-45-1 | aladdin |
| Valine | 72-18-4 | aladdin |
| Ornithine | 70-26-8 | aladdin |
| Beta-Alanine | 107-95-9 | aladdin |
| (2R)-2-Amino-3-Phosphonopropanoic Acid | 177120-10-8 | aladdin |
| Erythrose | 533-49-3 | aladdin |
| Galactose | 59-23-4 | aladdin |
| Ribitol | 488-81-3 | aladdin |
| Tryptophol | 526-55-6 | aladdin |
| Butyraldehyde | 123-72-8 | aladdin |
| Isoleucine | 73-32-5 | aladdin |
| 3-Cyanoalanine | 13538-04-8 | aladdin |
| Methionine | 63-68-3 | aladdin |
| L-Cysteine | 52-90-4 | aladdin |
| Tryptophan | 73-22-3 | aladdin |
| Saccharopine | 997-68-2 | aladdin |
| Methyl Trans-Cinnamate | 1754-62-7 | aladdin |
| 2-Hydroxy-3-Isopropylbutanedioic Acid | 3237-44-3 | aladdin |
| 6-Hydroxynicotinic Acid | 5006-66-6 | aladdin |
| 2,4-Diaminobutyric Acid | 161420-87-7 | aladdin |
| 4-Hydroxymandelic Acid | 184901-84-6 | aladdin |
| Homovanillic Acid | 306-08-1 | aladdin |
| Trans-3-Hydroxycinnamic Acid | 14755-02-3 | aladdin |
| 5-Hydroxyindole-3-Acetic Acid | 54-16-0 | aladdin |
| Elaidic Acid | 112-79-8 | aladdin |
| 6-Phosphogluconic Acid | 921-62-0 | aladdin |
| Phosphomycin | 78964-85-9 | aladdin |
| Fluorene | 86-73-7 | aladdin |
| Methyl-Beta-D-Galactopyranoside | 1824-94-8 | aladdin |
| Tetracosane | 646-31-1 | aladdin |
| Allose | 312-94-5 | aladdin |
| Sorbose | 87-79-6 | aladdin |
| Tagatose | 87-81-0 | aladdin |

Fig. S1. Sequence alignment comparison of B subunit of GerA germination receptor from *B. velezensis* SQR9 and *B. subtilis* NCIB 3610


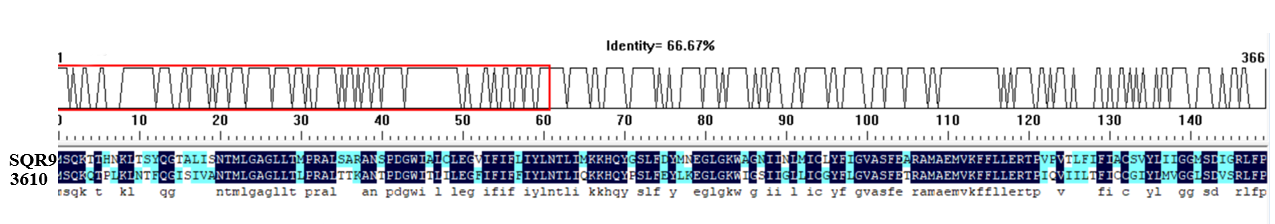

Supplement: Supplementary file 1 — Table S1: Bacillus strains used in this study. Table S2: Primers used for constructing and verifying strain of mutants and replenishment. Table S3: All chemicals used in the study. Figure S1: Sequence alignment comparison of the B subunit of the GerA germination receptor from B. velezensis SQR9 and B. subtilis NCIB 3610. [file MBT2-18-e70172-s001.docx]
